# An Insight into Perfusion Anisotropy within Solid Murine Lung Cancer Tumors

**DOI:** 10.3390/pharmaceutics16081009

**Published:** 2024-07-30

**Authors:** Antonio Martino, Rossana Terracciano, Bogdan Milićević, Miljan Milošević, Vladimir Simić, Blake C. Fallon, Yareli Carcamo-Bahena, Amber Lee R. Royal, Aileen A. Carcamo-Bahena, Edward Brian Butler, Richard C. Willson, Miloš Kojić, Carly S. Filgueira

**Affiliations:** 1 Department of Nanomedicine, Houston Methodist Research Institute, Houston, TX 77030, USA; amartino@houstonmethodist.org (A.M.); rossana.terracciano@polito.it (R.T.); bfallon@houstonmethodist.org (B.C.F.); ycarcamo@houstonmethodist.org (Y.C.-B.); alrroyal@houstonmethodist.org (A.L.R.R.); aileenayme@gmail.com (A.A.C.-B.); mkojic@houstonmethodist.org (M.K.); 2 Department of Materials Science and Engineering, University of Houston, Houston, TX 77024, USA; 3 Department of Electronics and Telecommunications, Politecnico di Torino, 10129 Torino, Italy; 4 Bioengineering Research and Development Center (BioIRC), 34000 Kragujevac, Serbia; bogdan.milicevic@uni.kg.ac.rs (B.M.); miljan.m@kg.ac.rs (M.M.); vladimir.simic.991@gmail.com (V.S.); 5 Faculty of Engineering, University of Kragujevac, 34000 Kragujevac, Serbia; 6 Institute for Information Technologies, University of Kragujevac, 34000 Kragujevac, Serbia; 7 Faculty of Information Technology, Belgrade Metropolitan University, 11000 Belgrade, Serbia; 8 Department of Radiation Oncology, Houston Methodist Research Institute, Houston, TX 77030, USA; ebutler@houstonmethodist.org; 9 Department of Chemical and Biomolecular Engineering, University of Houston, Houston, TX 77024, USA; willson@uh.edu; 10 Serbian Academy of Sciences and Arts, 11000 Belgrade, Serbia; 11 Department of Cardiovascular Surgery, Houston Methodist Research Institute, Houston, TX 77030, USA

**Keywords:** lung cancer, solid tumors, perfusion, vascularity, finite element computational model, smeared physical fields, Kojic Transport Model

## Abstract

Blood vessels are essential for maintaining tumor growth, progression, and metastasis, yet the tumor vasculature is under a constant state of remodeling. Since the tumor vasculature is an attractive therapeutic target, there is a need to predict the dynamic changes in intratumoral fluid pressure and velocity that occur across the tumor microenvironment (TME). The goal of this study was to obtain insight into perfusion anisotropy within lung tumors. To achieve this goal, we used the perfusion marker Hoechst 33342 and vascular endothelial marker CD31 to stain tumor sections from C57BL/6 mice harboring Lewis lung carcinoma tumors on their flank. Vasculature, capillary diameter, and permeability distribution were extracted at different time points along the tumor growth curve. A computational model was generated by applying a unique modeling approach based on the smeared physical fields (Kojic Transport Model, KTM). KTM predicts spatial and temporal changes in intratumoral pressure and fluid velocity within the growing tumor. Anisotropic perfusion occurs within two domains: capillary and extracellular space. Anisotropy in tumor structure causes the nonuniform distribution of pressure and fluid velocity. These results provide insights regarding local vascular distribution for optimal drug dosing and delivery to better predict distribution and duration of retention within the TME.

## 1. Introduction

New and innovative therapeutics, such as nanoscale-based pharmaceuticals, have rapidly advanced over the past few decades in cancer treatment, increasing the accumulation of antitumor agents in and around tumor tissues and improving their pharmacokinetics and release profiles, while reducing the dose to normal tissues [1]. Multifunctional platforms, such as organic or metallic nanoparticles, have been engineered to deliver therapeutic and diagnostic agents selectively to tumors [2], whereby tuning the size and surface properties of the nanostructure aids in overcoming physiological–pathological barriers to the treatment of drug-resistant cancers and facilitating drug penetration within malignant cells [3]. However, despite encouraging progress toward site-specific drug delivery [4,5] and the investigation of emerging technologies for sustained and local immunotherapeutic delivery [6,7,8,9], the extracellular matrix constitutes a major obstacle inhibiting therapeutic success and the mobility of diffusing species [10,11]. While efforts have been made to understand and model blood flow within the tumor and in vessels feeding tumors [12,13,14,15], there is still a need to predict changes in flow dynamics that occur across the tumor vessel network. Predicting changes in fluid dynamics surrounding and within the tumor would help to better screen for therapeutics capable of traversing the tumor microenvironment (TME).

One of the main reasons why current nanoscale-based pharmaceuticals fail to treat solid tumors lies within the high heterogeneity of the cancerous mass. In fact, the TME presents unique characteristics, including a high-density extracellular matrix (ECM), as well as abnormal tumor vasculature and lymphatic systems. Additionally, due to interstitial fibrosis and abnormal lymphatic vessels, the interstitial fluid pressure (IFP) in solid tumors increases to 5–130 mm Hg, compared to 0–3 mm Hg in healthy tissues [16]. All of these TME features hinder the transport of therapeutics into the tumor interstitium and its core area and, along with the rapid decrease in convection between the extravascular and intravascular spaces, create impenetrable barriers that ultimately result in ineffective drug doses to treat the target site [17]. Therefore, an enhancement in drug tumor penetration to achieve effective concentrations across the tumor mass is needed to improve therapeutic efficacy and advance novel and innovative therapeutics toward clinical translation.

To enhance the penetration and distribution of nanomaterials in solid tumors, efforts have been made to engineer the nanoparticle surface [18,19] for localized delivery of the particles to site-specific areas of the tumor [20,21]. For instance, several in vivo studies demonstrate that nanoparticles with phospholipid–polyethylene glycol-derived surface functionalization show significantly increased selective and efficient internalization by target cancer cells and tissues [22,23]. In addition, the physical and chemical properties of nanoparticles, such as particle size [24,25], morphology [26], and charge [27,28], can influence their delivery and distribution across the TME [29]. Moreover, various aspects of nanoparticles, such as passivation to prevent recognition and clearance [30], size and mass considerations for transport through biological barriers [12,14,31,32,33], and composition to enhance tumor accumulation based on patient-specific biomarkers [34], have all been studied to maximize drug delivery efficacy. While these efforts drive approaches toward personalized medicine, understanding TME dynamics is also critical.

Beyond considerations of particle properties to achieve better drug accumulation at the tumor site, computational and mathematical modeling of vascular flow and pressure across the tumor mass may also provide an opportunity to extract useful information for the targeted delivery of diagnostic and therapeutic agents [35,36]. In fact, theoretical models offer important tools for predicting and determining a range of parameters in physiologically relevant scenarios and testing potential solutions to overcome such biological barriers to reach target cells. For instance, a computational model by Frieboes et al. [37] effectively selected an optimal nanoparticle formulation in terms of particle size and vascular affinity to accumulate uniformly in the tumor mass as a function of the development stage of the malignancy. Stillman et al. [38] provided a modeling platform called the EVONANO, which is able to decrease both the time and cost required to develop nanoparticle designs by simulating tumor growth and nanoparticle transport using machine learning. However, these studies lack validation with both in vitro and in vivo experimental data. Guo et al. [39] described a quantitative metric system to identify and evaluate new cancer targets for tumor-targeting nanomedicines using comparative flow cytometric screening and characteristics, such as tumor specificity, target expression level, cellular internalization, therapeutic function, and potential clinical impact. However, although this methodology allows for the identification of parameters necessary for evaluating a potential therapeutic target to disrupt cancer pathogenesis, it was only tested in vitro with metastatic melanoma.

Computational models can also provide predictive information about therapeutic circulation and interactions with the tumor vasculature as well as tumor accumulation, payload release, efficacy, and safety [40]. For instance, Stapleton et al. [41] generated a mathematical model to describe pressure-driven fluid flow across blood vessels and through the tumor interstitium, extracting liposome accumulation curves from experimental computed tomography measurements in preclinical tumor models. A study by Caddy et al. [42] presented a three-part mathematical model to predict particle distribution after intratumoral injection, demonstrating that particles with a negative surface charge can spread farther from the injection location, occupying almost 90% more space when compared to particles with a neutral surface charge. However, this model was not validated experimentally. Finally, an in vivo biodistribution model was validated in work by Dogra et al. [43], whereby experimental data from the intravenous injection of mesoporous silica particles ranging in size from 46 to 162 nm into the plasma compartment of rats were used to develop a predictive mathematical model of whole-body nanoparticle pharmacokinetics and tumor delivery. The model was then validated with longitudinal in vivo data. Importantly, none of these models address parameters such as fluid velocity and intratumoral pressure, which can be useful predictors for developing strategies to target and retain therapeutics across the TME.

In this study, we investigated the biophysical properties of the TME, which plays a major role in fluid accumulation. Parameters including tumor vasculature and permeability were extracted from a pre-clinical study of lung cancer-bearing mice and the results were used to model tumor fluid transport dynamics. To generate a computational model of intratumoral perfusion, we implemented the finite element (FE) methodology based on smeared physical fields (termed the Kojic Transport Model, KTM), built in the FE program PAK-BIO (Kojic et al. [44]). We believe that our computational model will be useful for predicting therapeutic outcomes across different cancers, allowing for optimized drug carrier design and improved drug delivery efficiency and retention to advance personalized medicine.

## 2. Materials and Methods

### 2.1. Animal Model of Lung Cancer

In this study, we used six-week-old female C57BL/6 mice (*n* = 24, 6 per time point) to understand the conditions over time (vasculature, capillary diameter, and perfusion) in a mouse lung cancer model (Lewis lung carcinoma, LLC). These parameters were used to generate a theoretical model of intratumoral perfusion. The research protocol was granted Institutional Animal Care and Use Committee (IACUC) approval (protocol #IS00005178, approved 6 May 2019) at the Houston Methodist Research Institute. The animals were purchased from Taconic Biosciences (Rensselaer, NY, USA). Female mice were chosen for study because while both naive male and female C57BL/6 mice do not present lung function differences at baseline [45], tumors grow more rapidly in female C57BL/6 mice [46]. A Lewis lung carcinoma (LLC) cell line was used in this study as a murine model of non-small-cell lung cancer (NSCLC) since it is highly tumorigenic and provides a reproducible syngeneic model for lung cancer in the C57BL mouse.

### 2.2. Experimental Timeline of Perfusion Study

Under sedation, all mice received manual subcutaneous injection of 2 × 10^6^ LLC cells into their right flank. Mice weight (Figure S1A) and health conditions were monitored daily, ensuring adequate nutrients (food and water ad lib.) and living conditions (clean cages, enrichment). Tumor volumes were also measured daily using a digital caliper (McMaster-Carr, Elmhurst, IL, USA, 2340A11). Tumors were palpable 4–5 days after cell injection, and, after 10 days, tumor volumes reached an average of ~100 mm^3^. Then, 7, 10, 13, and 16 days post-tumor cell inoculation, *n* = 6/time point LLC tumor-bearing mice were administered the perfusion marker Hoechst 33342 (Thermo Fisher, Waltham, MA, USA, catalog number H3570) at a dose of 40 mg/kg intravenously through a lateral tail vein using insulin syringes (BD U 100 Insulin Syringe Micro Fine Needle 28G, Becton, Dickinson, Franklin Lakes, NJ, USA, 329461). This dose was chosen as it has previously been demonstrated to provide an apparent fluorescent signal [47]. We selected time points 7, 10, 13, and 16 days after LLC cell inoculation, identified as approximately 15%, 25%, 60%, and 80%, respectively, of final tumor growth, to strategically capture key stages of tumor development. The experimental endpoint was based on tumor volume greater than 2 cm^3^, tumor interfering with normal physiological function, surgical complications, or other symptoms as outlined in the HMRI Guidelines and Policies for Determination of Humane Endpoints and Tumor Monitoring Policy and the recommendations of the Comparative Medicine Program (CMP) for veterinary staff and was estimated to occur ~19 days after cell inoculation, representing 100% tumor growth. Tumor volumes (*V*) in mm^3^ were calculated through daily measurements of the tumor axes using digital calipers and the following formula:

(1) $$V=\frac{D*d^{2}}{2}$$

where *D* and *d* respectively represent the major and minor axes of the tumor measured in mm.

All animal procedures involving injections of cells were performed while the animals were under sedation, which was achieved by anesthetizing the mice with isoflurane. Isoflurane was administered as an inhalant at a dose of 2–5% for induction using an induction chamber and 1.5–2.5% for anesthetic maintenance using a nose cone throughout the injection procedure (<10 min) with a ~1.5–1.75 L per minute flow rate. For the Hoechst 33342 injection, the mice were positioned in commercially available collection platforms/restrainers, the skin was prepared using a gentle scrub of 70% isopropyl alcohol, gentle heat was applied to the tail for 30 s to 1 min to dilate the tail vein vessels, a 28 gauge needle attached to an appropriately sized syringe was inserted in the distal third of the tail at a parallel angle to the tail, and, once the vein had been punctured and a flash of blood was present in the needle hub, Hoechst 33342 (40 mg/kg) was slowly injected. Successful cannulation of the vessel resulted in “blanching” of the proximal lateral tail vein, and the needle was removed while gentle pressure was maintained on the puncture site. The animals were sacrificed one minute post-IV Hoechst 33342 injection via carbon dioxide asphyxiation, after which, euthanasia was verified. Tumors were excised (within ten minutes post-injection), weighed ex vivo (Figure S1B), and fixed in 10% formalin for further analysis.

### 2.3. Tumor Slice Preparation

After 24 h in formalin, each tumor was sectioned using a surgical blade and divided into two halves. This dissection procedure was performed by the same investigator for consistency. Each half-tumor was paraffin-embedded and then sectioned to generate a 5 μm medial slice.

### 2.4. Immunofluorescence (IF) Evaluations

Tumor morphology, vasculature (Rabbit anti-CD31 antibody at a 1:50 dilution; cat. no. ab28364; Abcam, Waltham, MA, USA; followed by a Goat anti-rabbit IgG (H + L) Cross-Adsorbed Secondary Antibody, Alexa Fluor™ 594 at a 1:200 dilution; cat. no. A-11012; Thermo Fisher Scientific, Waltham, MA, USA), and permeability (Hoechst 33342, Trihydrochloride, Trihydrate—FluoroPure Grade; cat. no. H21492; Invitrogen, Waltham, MA, USA) were assessed in tissue sections of LLC tumors.

### 2.5. Tumor 2D Imaging and Heatmaps

Tumor sections were imaged in their entirety using a SLIDEVIEW VS200 research slide scanner (Olympus, Center Valley, PA, USA) with a 10× objective. Separate images were obtained for Hoechst 33342 and CD31 staining using the OlyVia microscope software v3.4.1. Subsequently, the raw images were imported into ImageJ through the Bio-Formats plugin, where a 9 × 9 grid was systematically applied to each image. Image post-processing, involving background removal and the application of an over/under threshold with a minimum below setting of 79% and 95% for Hoeschst 33342 and CD31 images, respectively, was carried out in ImageJ to identify positively stained pixels.

For each tumor section, three distinct heatmaps were calculated, representing permeability (%), vasculature (%), and capillary diameter (µm). The permeability heatmaps illustrated the local density of pixels covered by Hoechst 33342 staining, expressed as the ratio of positively stained pixels to the total number of pixels in the grid cell. Vasculature heatmaps depicted the local density of pixels covered by CD31 staining, expressed as the ratio of positively stained pixels to the total number of pixels in the grid cell. Finally, capillary diameter heatmaps were determined through CD31 expression and quantified using ImageJ v1.54j, where measurements were performed across 90 to 318 different vessels per tumor section. Specifically, six stained capillaries were randomly selected and their diameter was measured and then averaged within each grid cell. Heatmaps were consistently generated for each tumor among the different selected time points (Figures S2–S5).

### 2.6. Computational Model

**Figure 1:**
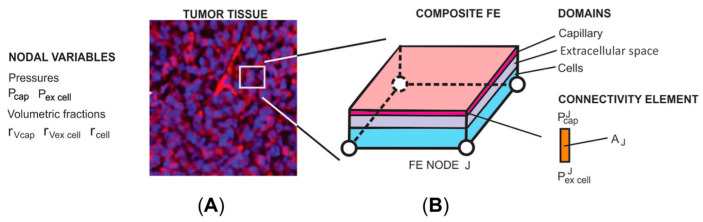
(**A**) Image (10×)of tumor tissue with capillary vessels stained using CD31 (red). (**B**) Composite smeared 2D finite element with 4 nodes and 3 physical domains: capillary, extracellular space, and cells. Fluid flow from the capillaries to the extracellular space is modeled by nodal connectivity elements, with the cross-sectional area equal to the surface (A_J_ in the figure).

Here, we briefly give basic information about the computational model. The FE model was generated by the implementation of the general concept based on the smeared physical fields (the KTM model). This methodology is adopted in several references [48,49,50,51,52,53,54,55,56] and it is well described and summarized by Kojic et al. [57,58]. The composite smeared 2D finite element used to model perfusion in a tumor is shown in Figure 1.

Fluid flow in the capillary and extracellular space is governed by Darcy’s law, which, in the FE format, has the following form (Kojic et al. [54]):

(2) $$K_{p}^{K}\Delta P^{K}=Q_{Vp}^{K}-K_{p}^{K}P^{K}$$

where *K* = 1 and *K* = 2 correspond to capillary and extracellular space, respectively, $P^{K}\;$ is the nodal pressure vector, $\Delta P^{K}$ are pressure increments in the time step, ${\;Q}_{Vp}^{K}$ is a volumetric term, and the FE matrix *K* is

(3) $$K_{\left(p\right)IJ}^{K(i-1)}=\int_{V}r_{V}^{K}k_{Dij}^{K}N_{I,i}N_{J,j}dV,\;\mathrm{sum}\;\mathrm{on}\;i,j:\;i,j=1,2$$

where $r_{V}^{K}$ represents the volumetric fractions of the capillary (*K* = 1) and extracellular space (*K* = 2). $k_{Dij}^{K}$ is the Darcy transport tensor (µm^2^/(Pa·s)) for lengths in µm, time in seconds, and pressure in Pa, which, for a capillary system, represents the tensor derived from the capillary geometry (directional cosines and diameters), while, for the tissue, it is a diagonal tensor of the Darcy coefficients. $N_{I,i}$ and $N_{J,j}$ are derivatives of the interpolation matrices and *V* is the element volume, which, for the 2D model used in this study, was equal to the element surface multiplied by the unit element thickness. Regarding the nodal connectivity elements, they were 1D elements (without axial dimension, i.e., they were fictitious elements) that connected the capillary and extracellular domains at each FE node. For FE node *j*, the balance equation in the form of Equation (2) was

(4) $$K_{ij}^{J}\Delta P_{j}^{J}=-K_{ij}^{J}P_{j}^{J},\;i,j=1,2;\;\mathrm{sum}\;\mathrm{on}\;j$$

where the conductivity matrix $K^{J}$ is

(5) $$K^{J}=\frac{4}{d_{cap}^{J}}r_{Vcap}^{J}V^{J}h_{cap}^{J}\left[\begin{array}{cc} 1 & -1\\ -1 & 1 \end{array}\right]$$

where $d_{cap}^{J},r_{Vcap}^{J},V^{J}$, and $h_{cap}^{J}$ are the capillary diameter, volumetric fraction of the capillary domain (where *K* = 1), volume of the continuum (representing a composite FE), and wall hydraulic permeability coefficient, all at node *J*, respectively. Since all quantities in the above expressions were assigned to nodes of the FE mesh, anisotropic characteristics of the capillary tissue system were considered in the computational model.

### 2.7. Statistical Analysis

All statistical analyses and graphs were obtained with GraphPad Prism (version 10.2.1; GraphPad Software, Inc., San Diego, CA, USA). Mean ± s.e.m. values were calculated and plotted in the results. The comparison of means between different groups of numerical variables was performed using a one-way ANOVA, where *p* values (* *p* < 0.05, ** *p* < 0.01) were considered statistically significant. Figures of pressure and velocity fields along with diagrams were generated using our in-house CAD software (https://github.com/miljanmilos/CAD-Solid-Field, accessed on 24 July 2024). Pressure and velocity values were obtained using measured data and an inverse-distance weighting interpolation procedure. Also, linear interpolation of geometry and other data was adopted for time points (time steps of the computational model) between the three data points.

## 3. Results

### 3.1. Experimental Results

**Figure 2:**
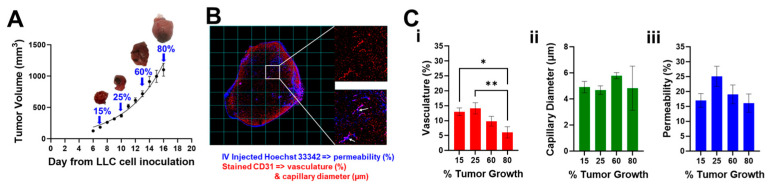
Assessments of tumor vasculature and permeability in a lung cancer mouse model. (**A**) LLC tumor volume growth curve. Blue arrows indicate 15% (183.4 ± 11.7), 25% (365.7 ± 23.0 mm^3^), 60% (717.2 ± 47.0 mm^3^), and 80% (1100.1 ± 102.8 mm^3^) of the curve where tumors were dissected after Hoechst 33342 IV injections (permeability) and CD31 (vasculature and capillary diameter) staining. (**B**) Representative fluorescence tumor image (10×) at 15% tumor growth: Hoechst (blue), CD31 (red). Insets show microvessels stained within the tumor (top, red channel only) and IV perfused Hoechst 33342 dye, which could be visualized by a halo of blue fluorescence (white arrows) near the vessels (bottom, red and blue channels). Data are plotted and reported as mean ± s.e.m. values. (**C**) Quantification of (**i**) vasculature, (**ii**) capillary diameter, and (**iii**) permeability over time (* *p* < 0.05 and ** *p* < 0.01). Data in (**A**) were fit using the exponential growth curve equation y = 59.98 × exp (0.1874x).

Figure 2A shows the growth curve of tumor volumes measured manually by an external caliper, as described in Section 2.2. Tumors at 15%, 25%, 60%, and 80% of the final volume over the growth curve are indicated with a blue arrow and a representative photo of the tumor is presented. Figure 2B shows a representative fluorescence image of an entire tumor slice with overlapped channels for Hoechst (blue) and CD31 (red) as well as insets of two different grid cells. Hoechst 33342 was used specifically for staining the lung cancer cell nuclei and CD31 was used as a microvasculature marker. CD31 showed evidence of microvessels across the tumor (top inset, red channel only) and Hoechst 33342 could be visualized by a halo of blue fluorescence near the vessels (bottom inset, red and blue channels). Despite the short half-life of Hoechst 33342 [47], intravenous injections resulted in visible fluorescence across all the animals (Figures S2–S5, left columns). Figure 2C shows graphs of quantified (i) vasculature, (ii) capillary diameter, and (iii) permeability over time. While the percent perfused area and capillary diameter changes were not significant as tumor growth increased, we did observe significant changes in the percent vasculature (* *p* < 0.05 between 15% and 80% and ** *p* < 0.01 between 25% and 80%). Early increases in vasculature density may be attributable to tumor angiogenesis [59], while later decreases may be attributable to necrosis [60].

**Figure 3:**
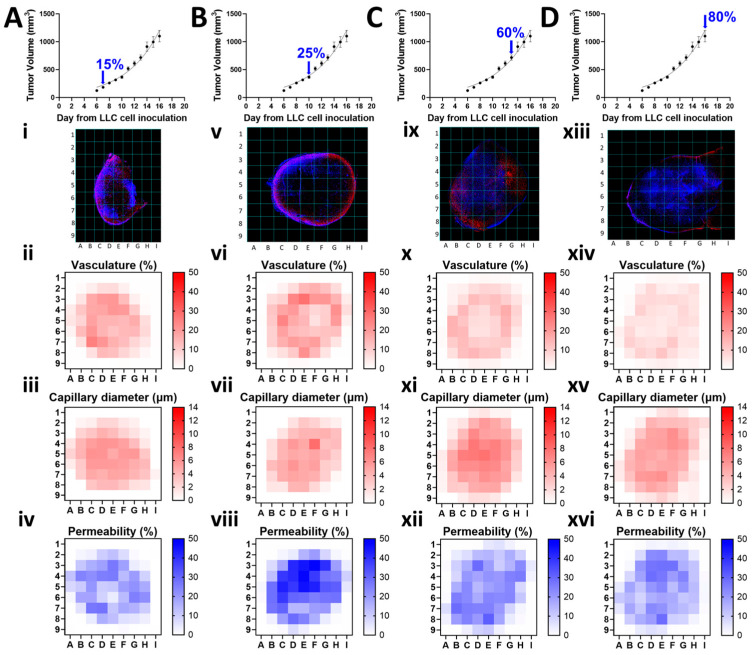
Vasculature (%), capillary diameter (µm), and permeability (%) distribution at different time points along the tumor growth curve. Tumor volume at (**A**) 15%, (**B**) 25%, (**C**) 60%, and (**D**) 80% of total growth. Representative fluorescent maps showing co-expression of Hoechst 33342 (blue) and CD31 (red) across sliced tumor area (**i**,**v**,**ix**,**xiii**). Heat maps showing local changes in the average vasculature (**ii**,**vi**,**x**,**xiv**) and capillary diameter (**iii**,**vii**,**xi**,**xv**) determined by CD31 expression and average permeability (**iv**,**viii**,**xii**,**xvi**) determined by Hoechst 33342 expression across the different time points.

To examine the local and temporal changes in tumor structure, we calculated the % vasculature, average capillary diameter in µm, and % permeability at different time points along the tumor growth curve by applying a grid on each fluorescence image, removing the background through thresholding, quantifying the signal intensity (vasculature and permeability) or average vessel (capillary) diameter on each grid cell, and plotting them individually as heatmaps. Figure 3 illustrates this methodology, where each column represents a distinct time point corresponding to percent tumor growth at 15% (A), 25% (B), 60% (C), and 80% (D). Within each column, a representative fluorescence image of a mouse tumor specimen is accompanied by heatmaps depicting average vasculature, capillary diameter, and permeability for that time point. The heatmaps reveal changes in vasculature (ii, vi, x, xiv), capillary diameter (iii, vii, xi, xv), and permeability (iv, viii, xii, xvi) across the four different time points. Additional grid images and heatmaps for each tumor sample are provided in Supplemental Figures S2–S5. By visualizing these parameters as individual heatmaps, local information was spatially preserved that would otherwise have been lost by sample averaging.

### 3.2. Computational Results

Here, we briefly outline the computational procedure steps for the FE model generation. The computational model was generated from images shown in Figure 3i,v,ix,xiii (and Supplemental Figures S2–S5), where the tumor domain is divided into a 9 × 9 mesh. We imposed a constant pressure of 10 mmHg within capillaries, which took into account hydrostatic and oncotic pressures and arteriolar and venular participation within the capillary network [61,62,63,64]. This pressure was relevant for the fluid transport from capillaries to the extracellular space within the tumor. Also, following the same data [64], we imposed zero pressure at the contour of the model as the boundary condition, assuming that there was a balance between perfusion and reabsorption between capillaries and tissue extracellular space. Using Figure S2i–vi, we extracted the contours from each of the six tumors at 15% total tumor growth and calculated an average geometry, which was used to set an initial configuration for our simulation. We then extracted contours from each of the tumors from Figures S3i–vi, S4i–vi, and S5i–vi and determined average geometries corresponding to 25%, 60%, and 80% of the total growth. During finite element simulation, we linearly interpolated boundary contours (for both geometric shape and size) between each experimental time point based on acquired average configurations.

Next, we applied the experimentally averaged vasculature, capillary diameter, and permeability to our model (Figure 3ii–iv,vi–viii,x–xii,xiv–xvi) in Equations (3) and (5). In this process, to assign all the data, equation solutions were interpolated using the inverse of the distance between two nodes as a weighting factor in accordance with the position of the final element node within the averaged heatmaps. Within each time point of the finite element computation, re-meshing was performed, allowing for finite elements not to be fixed in space by their size or shape, since the geometry evolved during the calculations. Evaluation of the capillary volumetric fraction *r_Vcap_* was performed as follows (described here for the parameters at 15% of the tumor growth, represented as the first column in Figure 3, but broadly applicable to any time point and any node J of the FE model). The averaged vasculature displayed in Figure 3ii was first assumed to be equivalent to the capillary internal surface (*A_cap_*) divided by the total surface of the cell grid (*A_tot_*) to yield a percentage ($A_{cap}^{\%}$), where $A_{cap}^{\%}=100A_{cap}/A_{tot}$, and then expressed as

(6) $$A_{cap}^{\%}=\frac{100A_{cap}}{A_{tot}}=\frac{100d\pi L}{A_{tot}}$$

to provide results in terms of capillary diameter (*d*), shown in Figure 3iii, and capillary length (*L*). Since capillary volume could be expressed as *Vcap* = *d*^2^*πL*/4, we could then obtain capillary volumetric fraction *r_vcap_* at a point of the surface as

(7) $$r_{Vcap}=\frac{V_{cap}}{A_{tot}h_{z}}=\frac{d}{400h_{z}}A_{cap}^{\%}$$

where *h_z_* = 1 µm is the model thickness in the direction normal to the plane (Supplemental Figure S6). Evaluation of the volumetric fraction of the extracellular space (*r_ex_*) was performed as follows. The average permeability displayed in Figure 3iv was assumed to be equivalent to the area covered by the cells (*A_cell_*) divided by the total surface of the cell grid (*A_tot_*) to yield cell volumetric fraction (*r_vcell_*), where *r_Vcell_* = *A_cell_*/*A_tot_*. We then expressed the volumetric fraction of the extracellular space (*r_ex_*) as

(8) $$r_{ex}=1-r_{Vcap}-r_{Vcell}$$

Finally, the wall hydraulic coefficient $h_{cap}^{J}\;$in Equation (5) was obtained from the filtration coefficient (*Kf*) in reference [63], reduced to the unit surface, and expressed as 1.57 × 10^−3^ μm/(Pa·s). We included perfusion anisotropy within the tumor by Equations (7) and (8) since the volumetric fractions of the capillaries and extracellular space varied over the model domain in accordance with our experimental records.

**Figure 4:**
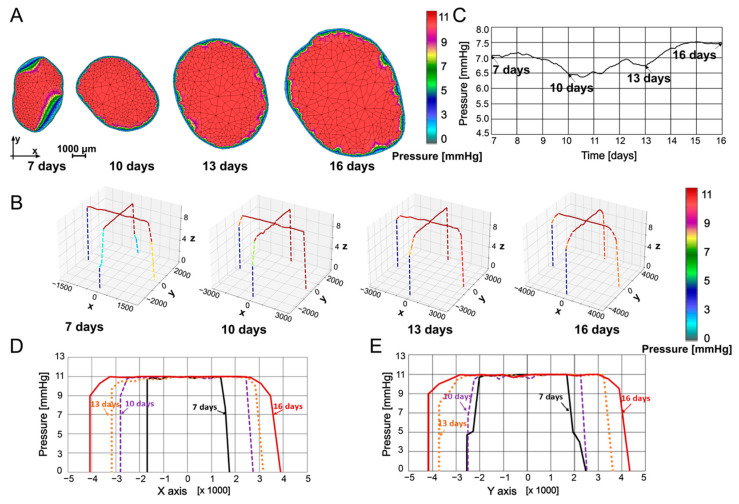
(**A**) Pressure field at 7, 10, 13, and 16 days. (**B**) Pressure field in a 3D representation at 7, 10, 13, and 16 days. (**C**) Mean pressure vs time. (**D**) Pressure distribution along *x*-axis. (**E**) Pressure distribution along *y*-axis.

**Figure 5:**
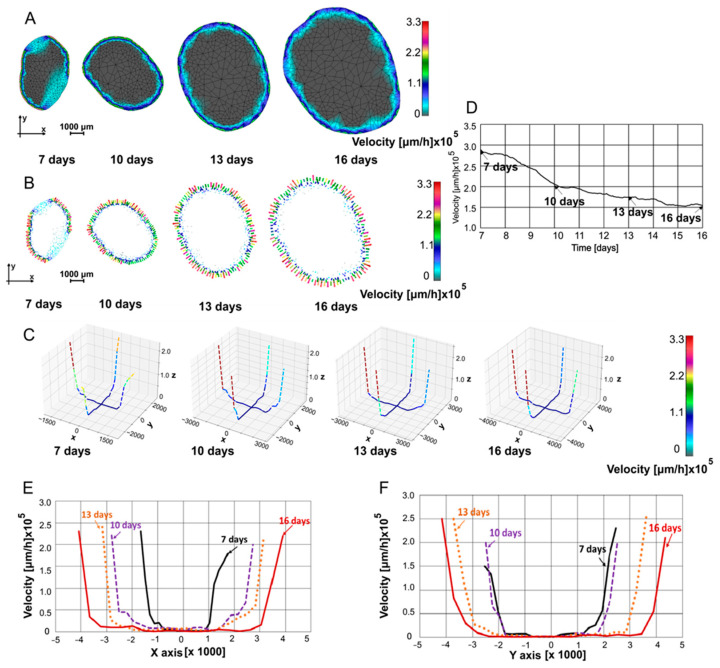
(**A**) Velocity field at 7, 10, 13, and 16 days. (**B**) Vector velocity field at 7, 10, 13, and 16 days. (**C**) Velocity field in 3D representation at 7, 10, 13, and 16 days. (**D**) Mean velocity modulus vs. time. (**E**) Velocity distribution along *x*-axis. (**F**) Velocity distribution along *y*-axis.

In Figure 4, pressures within the tumor at 7, 10, 13, and 16 days are presented. Pressures were determined through interpolation from the previous time point’s mesh to the subsequent time point’s mesh, ensuring that all necessary values for finite element analysis were available at the current time point. Figure 4A shows the pressure maps obtained using our in-house CAD software and finite element analysis, while, in Figure 4B, pressure values are plotted in a 3D representation along coordinate axes x and y at the tumor center. Maximum pressure values did not vary much over the specified period. Some increase in the mean pressure was due to increases in the size of the domain with high pressure relative to the domain close to the boundary, where the pressure gradient was leaning to the zero value. The highest values were distributed along the model, starting from the center of the tumor and spreading to the boundary. These results were in qualitative agreement with those reported in [65]. In Figure 4C, the mean pressure values are plotted over time for insight into pressure evolution, while the spatial distribution of the pressure is displayed in Figure 4D,E. Since the model required geometries as well as input and output boundaries, we chose to present the data in Figure 4C and Figure 5D with an *x*-axis starting at 7 days (15%) and ending at 16 days (80%). However, a linear interpolation could be performed for time points prior to 7 days as well as after 16 days, but performing this interpolation required a geometry and growth trend set equal to the adjacent interval, which was an assumption not observed experimentally, and, therefore, imprecise. In both the x and y directions, pressure was the highest in the middle of the tumor, decreasing to zero at the tumor boundary. It can be seen from Figure 4C–E that pressure nonuniformly changed over time, with the values at 16 days increased with respect to the 7-day configuration.

In Figure 5, fluid velocity fields at 7, 10, 13, and 16 days are shown. Velocity fields were determined through interpolation from the previous time point’s mesh to the subsequent time point’s mesh, ensuring that all necessary values for finite element analysis were available at the current time point. Figure 5A displays the velocity maps generated using our in-house CAD software and finite element analysis, while, in Figure 5B, velocities in the form of vectors are shown. In Figure 5C, velocity values are plotted in a 3D representation along the same two selected lines as in Figure 4B. It can be seen that as the tumor grew, the mean velocities slightly decreased. The velocities were always the largest at the boundary of the tumor since the pressure gradients were maximal at the boundary. In Figure 5D, the mean velocity modulus values are plotted over time to obtain insight into overall velocity values over time. The nonlinear decrease of the mean velocity was due to the increase in the domain with small velocities relative to the domain close to the boundary where the high pressure gradient led to high velocity values. For the spatial distribution along the *x*-axis, the velocities at the boundary first increased as the tumor grew but then decreased (Figure 5E). Along the *y*-axis, the velocities at the boundary increased over the observed period (Figure 5F), illustrating perfusion anisotropy within the tumor.

## 4. Discussion

As tumor microenvironment complexity compromises therapeutic delivery, tumor-specific biophysical properties must be evaluated as a dynamic tumor growth process. This study aimed to predict intratumoral fluid pressure and velocity in three-dimensional space over time, allowing for coordinated temporal and spatial delivery of fluids in the intratumoral space. We used a pre-clinical model of lung cancer to generate images of tumor cross-sections and generated a computational model based on the smeared physical fields (Kojic Transport Model, KTM) to determine the distribution of pressure and velocity within the tumor during its growth. A total of 7, 10, 13, and 16 days after tumor cell inoculation, tumor-bearing mice were intravenously administered the perfusion marker Hoechst 33342 (40 mg/kg) immediately prior to euthanasia. Tumor sections were stained with the endothelial marker CD31 for vascular detection and imaged in their entirety. Image postprocessing was performed to extract vasculature, capillary diameter, and permeability distribution at different time points along the tumor growth curve. Notably, it was observed that at earlier time points along the tumor growth curve (<25%), there was an increase in vascular density, which may be attributed to changes in angiogenesis, while, at later time points, there was a significant decrease in percent vasculature (* *p* < 0.05 between 15% and 80% and ** *p* < 0.01 between 25% and 80%), which may be attributed to necrosis in the center of advanced tumors. Necrosis and collagen content affect transport characteristics within tissue [15]. We modeled fluid flow in the capillary and extracellular space using a composite smeared 2D finite element. Outcomes from the model included fluid velocity and pressure over time as well as the spatial distribution of velocity and pressure. Others have shown that IFP changes across the tumor and can reach maximum values near the tumor center, with microvascular pressure as the main driving force [65], and that when limiting factors for the transport of biologically useful macromolecules are known, specific measures may be taken to circumvent such difficulties [66]. We also found with the KTM model that the highest pressure values resided near the center of the tumor and reduced in strength close to the tumor boundary, while velocities were highest at the tumor boundary but illustrated spatial perfusion anisotropy when evaluated across time. This model was the first attempt to obtain insight into an anisotropic view of pressure distribution in the tumor. While the subject here was perfusion, importantly, the model can be extended to study drug transport by diffusion [57].

However, our study presents several limitations. First, the experimental sample size was not sufficient to highlight statistical differences in vasculature and perfusion rates over time due to the increased margin of error. Second, given that the capillary beds were numerous and highly complex [48] and precise information about capillary diameter and network geometry was confined to small areas of capture, extrapolations were necessary to larger regions to implement the model. Although our KTM model did not require the modeling of each capillary as a 1D structure, data for evaluation of the transport tensor (Kojic et al. [58]) within Equation (3) were needed, such that the evaluated transport tensor could be interpolated over the model domain. Third, while the KTM model can support the generation of a 3D model using stacked imaging providing information on flow patterns in the z-plane, this would require extensive tumor slicing and imaging. Therefore, we present a 2D finite element model based on the assumption that the flow pattern is the same for all layers parallel to the considered x,y plane (Supplemental Figure S6). Despite these limitations, we demonstrate the robustness of the Kojic Transport Model and its applicability across a real physiological condition, solid lung tumor growth. Information extracted from this model regarding fluid velocity and pressure could prove useful in characterizing a tumor’s metabolic profile as well as distinguishing whether a tumor would prove amenable to certain types of therapies (i.e., radiation, chemo-, and/or immune therapy).

## 5. Conclusions

In summary, we investigated tumor-specific biophysical properties during a dynamic tumor growth process and used experimental data to generate a mathematical model that predicted intratumoral fluid pressure and velocity in a three-dimensional space over time. Further evaluation will expand our knowledge of the tumor microenvironment to better predict therapeutic outcomes across different cancer subtypes, leading to optimized drug carrier design and more effective dosing regimens, thereby offering a more personalized approach to medicine.

## Data Availability

The executable of our preprocessing and post-processing software for finite element modeling, along with the executable of our finite element analysis software, can be found in the GitHub repository https://github.com/miljanmilos/CAD-Solid-Field accessed 22 September 2022.

## Supplementary Materials

The following supporting information can be downloaded at https://www.mdpi.com/article/10.3390/pharmaceutics16081009/s1: Figure S1: (A) Mice and (B) ex vivo tumor weights. Dotted line (grey) represents the time point for cell inoculation (day 0). Dashed lines (blue) represent tumor harvest for the 15% group (day 7), 25% group (day 10), 60% group (day 13), and 80% group (day 16); Figure S2: Fluorescence maps showing co-expression of Hoechst 33342 (blue) and CD31 (red) across sliced tumor area (i–vi) at 15% tumor growth for six different samples. Matrix size (width and height) are reported for each image. Heat maps showing changes in (A, D, G, J, M, P) vasculature determined by CD31 expression, (B, E, H, K, N, Q) capillary diameter determined by CD31 expression, and (C, F, I, L, O, R) permeability determined by Hoechst 33342 expression; Figure S3: Fluorescence maps showing co-expression of Hoechst 33342 (blue) and CD31 (red) across sliced tumor area (i–vi) at 25% tumor growth for six different samples. Matrix size (width and height) are reported for each image. Heat maps showing changes in (A, D, G, J, M, P) vasculature determined by CD31 expression, (B, E, H, K, N, Q) capillary diameter determined by CD31 expression, and (C, F, I, L, O, R) permeability determined by Hoechst 33342 expression; Figure S4: Fluorescence maps showing co-expression of Hoechst 33342 (blue) and CD31 (red) across the sliced tumor area (i–vi) at 60% tumor growth for six different samples. Matrix size (width and height) are reported for each image. Heat maps showing changes in (A, D, G, J, M, P) vasculature determined by CD31 expression, (B, E, H, K, N, Q) capillary diameter determined by CD31 expression, and (C, F, I, L, O, R) permeability determined by Hoechst 33342 expression; Figure S5: Fluorescence maps showing co-expression of Hoechst 33342 (blue) and CD31 (red) across sliced tumor area (i–vi) at 80% tumor growth for six different samples. Matrix size (width and height) are reported for each image. Heat maps showing changes in (A, D, G, J, M, P) vasculature determined by CD31 expression, (B, E, H, K, N, Q) capillary diameter determined by CD31 expression, and (C, F, I, L, O, R) permeability determined by Hoechst 33342 expression; Figure S6: A 3D representation based on the assumption that the flow pattern is consistent across all layers parallel to the x, y plane of the 2D model.

## Abbreviations

Area covered by the cells	A_cell_
Capillary diameter	$d_{cap}^{J}$
Capillary internal surface	A_cap_
Capillary length	L
Capillary volumetric fraction	r_vcap_
Cell volumetric fraction	r_vcell_
Comparative Medicine Program	CMP
Darcy coefficients	N_I,i_ and N_J,j_
Pressure increments in time step	ΔP^K^
Extracellular matrix	ECM
Finite element	FE
FE matrix	K
Filtration coefficient	Kf
Hydraulic coefficient	$h_{cap}^{J}$
Immunofluorescence	IF
Institutional Animal Care and Use Committee	IACUC
Interstitial fluid pressure	IFP
Kojic Transport Model	KTM
Lewis lung carcinoma	LLC
Nodal pressure vector	P^K^
Non-small-cell lung cancer	NSCLC
Total surface of the cell grid	*A_tot_*
Tumor microenvironment	TME
Tumor volumes	V
Volume of the continuum	$V^{J}$
Volumetric fraction	$r_{V}^{K}$
Volumetric fraction of the capillary domain	$r_{Vcap}^{J}$
Volumetric fraction of the extracellular space	r_ex_
Volumetric term	$Q_{Vp}^{K}$
Wall hydraulic permeability coefficient	$h_{cap}^{J}$

## References

[1] Rodríguez F., Caruana P., De la Fuente N., Español P., Gámez M., Balart J., Llurba E., Rovira R., Ruiz R., Martín-Lorente C. (2022). Nano-Based Approved Pharmaceuticals for Cancer Treatment: Present and Future Challenges. Biomolecules.

[2] Terracciano R., Demarchi D., Ruo Roch M., Aiassa S., Pagana G. (2021). Nanomaterials to Fight Cancer: An Overview on Their Multifunctional Exploitability. J. Nanosci. Nanotechnol..

[3] Li Y., Xu X. (2020). Nanomedicine Solutions to Intricate Physiological-Pathological Barriers and Molecular Mechanisms of Tumor Multidrug Resistance. J. Control. Release.

[4] Anselmo A.C., Mitragotri S. (2019). Nanoparticles in the Clinic: An Update. Bioeng. Transl. Med..

[5] Choi M.-R., Stanton-Maxey K.J., Stanley J.K., Levin C.S., Bardhan R., Akin D., Badve S., Sturgis J., Robinson J.P., Bashir R. (2007). A Cellular Trojan Horse for Delivery of Therapeutic Nanoparticles into Tumors. Nano Lett..

[6] Liu H.-C., Viswanath D.I., Pesaresi F., Xu Y., Zhang L., Di Trani N., Paez-Mayorga J., Hernandez N., Wang Y., Erm D.R. (2021). Potentiating Antitumor Efficacy through Radiation and Sustained Intratumoral Delivery of Anti-CD40 and Anti-PDL1. Int. J. Radiat. Oncol. Biol. Phys..

[7] Viswanath D.I., Liu H.-C., Huston D.P., Chua C.Y.X., Grattoni A. (2022). Emerging Biomaterial-Based Strategies for Personalized Therapeutic in Situ Cancer Vaccines. Biomaterials.

[8] Chua C.Y.X., Ho J., Susnjar A., Lolli G., Di Trani N., Pesaresi F., Zhang M., Nance E., Grattoni A. (2020). Intratumoral Nanofluidic System for Enhancing Tumor Biodistribution of Agonist CD40 Antibody. Adv. Therap..

[9] Chua C.Y.X., Jain P., Susnjar A., Rhudy J., Folci M., Ballerini A., Gilbert A., Singh S., Bruno G., Filgueira C.S. (2018). Nanofluidic Drug-Eluting Seed for Sustained Intratumoral Immunotherapy in Triple Negative Breast Cancer. J. Control. Release.

[10] Tomasetti L., Breunig M. (2018). Preventing Obstructions of Nanosized Drug Delivery Systems by the Extracellular Matrix. Adv. Healthc. Mater..

[11] Lee B.J., Cheema Y., Bader S., Duncan G.A. (2021). Shaping Nanoparticle Diffusion through Biological Barriers to Drug Delivery. JCIS Open.

[12] Nizzero S., Ziemys A., Ferrari M. (2018). Transport Barriers and Oncophysics in Cancer Treatment. Trends Cancer.

[13] van de Ven A.L., Wu M., Lowengrub J., McDougall S.R., Chaplain M.A.J., Cristini V., Ferrari M., Frieboes H.B. (2012). Integrated Intravital Microscopy and Mathematical Modeling to Optimize Nanotherapeutics Delivery to Tumors. AIP Adv..

[14] Ferrari M. (2010). Frontiers in Cancer Nanomedicine: Directing Mass Transport through Biological Barriers. Trends Biotechnol..

[15] Yokoi K., Kojic M., Milosevic M., Tanei T., Ferrari M., Ziemys A. (2014). Capillary-Wall Collagen as a Biophysical Marker of Nanotherapeutic Permeability into the Tumor Microenvironment. Cancer Res..

[16] Heldin C.-H., Rubin K., Pietras K., Ostman A. (2004). High Interstitial Fluid Pressure—An Obstacle in Cancer Therapy. Nat. Rev. Cancer.

[17] Niu Y., Zhu J., Li Y., Shi H., Gong Y., Li R., Huo Q., Ma T., Liu Y. (2018). Size Shrinkable Drug Delivery Nanosystems and Priming the Tumor Microenvironment for Deep Intratumoral Penetration of Nanoparticles. J. Control. Release.

[18] Terracciano R., Zhang A., Butler E.B., Demarchi D., Hafner J.H., Grattoni A., Filgueira C.S. (2021). Effects of Surface Protein Adsorption on the Distribution and Retention of Intratumorally Administered Gold Nanoparticles. Pharmaceutics.

[19] Terracciano R., Sprouse M.L., Wang D., Ricchetti S., Hirsch M., Ferrante N., Butler E.B., Demarchi D., Grattoni A., Filgueira C.S. Intratumoral Gold Nanoparticle-Enhanced CT Imaging: An in Vivo Investigation of Biodistribution and Retention. Proceedings of the 2020 IEEE 20th International Conference on Nanotechnology (IEEE-NANO).

[20] Terracciano R., Carcamo-Bahena Y., Royal A.L.R., Messina L., Delk J., Butler E.B., Demarchi D., Grattoni A., Wang Z., Cristini V. (2022). Zonal Intratumoral Delivery of Nanoparticles Guided by Surface Functionalization. Langmuir.

[21] Terracciano R., Carcamo-Bahena Y., Butler E.B., Demarchi D., Grattoni A., Filgueira C.S. (2021). Hyaluronate-Thiol Passivation Enhances Gold Nanoparticle Peritumoral Distribution When Administered Intratumorally in Lung Cancer. Biomedicines.

[22] Zhang D., Zhang J. (2020). Surface Engineering of Nanomaterials with Phospholipid-Polyethylene Glycol-Derived Functional Conjugates for Molecular Imaging and Targeted Therapy. Biomaterials.

[23] Tian H., Zhang T., Qin S., Huang Z., Zhou L., Shi J., Nice E.C., Xie N., Huang C., Shen Z. (2022). Enhancing the Therapeutic Efficacy of Nanoparticles for Cancer Treatment Using Versatile Targeted Strategies. J. Hematol. Oncol..

[24] Perry J.L., Reuter K.G., Luft J.C., Pecot C.V., Zamboni W., DeSimone J.M. (2017). Mediating Passive Tumor Accumulation through Particle Size, Tumor Type, and Location. Nano Lett..

[25] Pandey A., Vighetto V., Di Marzio N., Ferraro F., Hirsch M., Ferrante N., Mitra S., Grattoni A., Filgueira C.S. (2020). Gold Nanoparticles Radio-Sensitize and Reduce Cell Survival in Lewis Lung Carcinoma. Nanomaterials.

[26] Wang Z., Wu Z., Liu J., Zhang W. (2018). Particle Morphology: An Important Factor Affecting Drug Delivery by Nanocarriers into Solid Tumors. Expert Opin. Drug Deliv..

[27] Held K.D., Kawamura H., Kaminuma T., Paz A.E.S., Yoshida Y., Liu Q., Willers H., Takahashi A. (2016). Effects of Charged Particles on Human Tumor Cells. Front. Oncol..

[28] Terracciano R., Zhang A., Simeral M.L., Demarchi D., Hafner J.H., Filgueira C.S. (2021). Improvements in Gold Nanorod Biocompatibility with Sodium Dodecyl Sulfate Stabilization. J. Nanotheranostics.

[29] Zhang M., Gao S., Yang D., Fang Y., Lin X., Jin X., Liu Y., Liu X., Su K., Shi K. (2021). Influencing Factors and Strategies of Enhancing Nanoparticles into Tumors in Vivo. Acta Pharm. Sin. B.

[30] Ferrari M. (2005). Cancer Nanotechnology: Opportunities and Challenges. Nat. Rev. Cancer.

[31] Ziemys A., Kojic M., Milosevic M., Ferrari M. (2012). Interfacial Effects on Nanoconfined Diffusive Mass Transport Regimes. Phys. Rev. Lett..

[32] Ziemys A., Kojic M., Milosevic M., Kojic N., Hussain F., Ferrari M., Grattoni A. (2011). Hierarchical Modeling of Diffusive Transport through Nanochannels by Coupling Molecular Dynamics with Finite Element Method. J. Comput. Phys..

[33] Blanco E., Ferrari M. (2014). Emerging Nanotherapeutic Strategies in Breast Cancer. Breast.

[34] Yokoi K., Tanei T., Godin B., van de Ven A.L., Hanibuchi M., Matsunoki A., Alexander J., Ferrari M. (2014). Serum Biomarkers for Personalization of Nanotherapeutics-Based Therapy in Different Tumor and Organ Microenvironments. Cancer Lett..

[35] Liu Y., Shah S., Tan J. (2012). Computational Modeling of Nanoparticle Targeted Drug Delivery. Rev. Nanosci. Nanotechnol..

[36] Kaddi C.D., Phan J.H., Wang M.D. (2013). Computational Nanomedicine: Modeling of Nanoparticle-Mediated Hyperthermal Cancer Therapy. Nanomedicine.

[37] Frieboes H.B., Wu M., Lowengrub J., Decuzzi P., Cristini V. (2013). A Computational Model for Predicting Nanoparticle Accumulation in Tumor Vasculature. PLoS ONE.

[38] Stillman N.R., Balaz I., Tsompanas M.-A., Kovacevic M., Azimi S., Lafond S., Adamatzky A., Hauert S. (2021). Evolutionary Computational Platform for the Automatic Discovery of Nanocarriers for Cancer Treatment. npj Comput. Mater..

[39] Guo P., Yang J., Bielenberg D.R., Dillon D., Zurakowski D., Moses M.A., Auguste D.T. (2017). A Quantitative Method for Screening and Identifying Molecular Targets for Nanomedicine. J. Control. Release.

[40] Kutumova E.O., Akberdin I.R., Kiselev I.N., Sharipov R.N., Egorova V.S., Syrocheva A.O., Parodi A., Zamyatnin A.A., Kolpakov F.A. (2022). Physiologically Based Pharmacokinetic Modeling of Nanoparticle Biodistribution: A Review of Existing Models, Simulation Software, and Data Analysis Tools. Int. J. Mol. Sci..

[41] Stapleton S., Milosevic M., Allen C., Zheng J., Dunne M., Yeung I., Jaffray D.A. (2013). A Mathematical Model of the Enhanced Permeability and Retention Effect for Liposome Transport in Solid Tumors. PLoS ONE.

[42] Caddy G., Stebbing J., Wakefield G., Xu X.Y. (2022). Modelling of Nanoparticle Distribution in a Spherical Tumour during and Following Local Injection. Pharmaceutics.

[43] Dogra P., Butner J.D., Ruiz Ramírez J., Chuang Y., Noureddine A., Jeffrey Brinker C., Cristini V., Wang Z. (2020). A Mathematical Model to Predict Nanomedicine Pharmacokinetics and Tumor Delivery. Comput. Struct. Biotechnol. J..

[44] Kojic M., Filipovic N., Milosevic M. (2020). PAK-BIO.

[45] Card J.W., Carey M.A., Bradbury J.A., DeGraff L.M., Morgan D.L., Moorman M.P., Flake G.P., Zeldin D.C. (2006). Gender Differences in Murine Airway Responsiveness and Lipopolysaccharide-Induced Inflammation. J. Immunol..

[46] Thompson M.G., Peiffer D.S., Larson M., Navarro F., Watkins S.K. (2017). FOXO3, Estrogen Receptor Alpha, and Androgen Receptor Impact Tumor Growth Rate and Infiltration of Dendritic Cell Subsets Differentially between Male and Female Mice. Cancer Immunol. Immunother..

[47] Smith K.A., Hill S.A., Begg A.C., Denekamp J. (1988). Validation of the Fluorescent Dye Hoechst 33342 as a Vascular Space Marker in Tumours. Br. J. Cancer.

[48] Kojic M., Milosevic M., Simic V., Koay E.J., Fleming J.B., Nizzero S., Kojic N., Ziemys A., Ferrari M. (2017). A Composite Smeared Finite Element for Mass Transport in Capillary Systems and Biological Tissue. Comput. Methods Appl. Mech. Eng..

[49] Kojic M., Milosevic M., Simic V., Koay E.J., Kojic N., Ziemys A., Ferrari M. (2017). Extension of the Composite Smeared Finite Element (CSFE) to Include Lymphatic System in Modeling Mass Transport in Capillary Systems and Biological Tissue. J. Serbian Soc. Comput. Mech..

[50] Kojic M., Simic V., Milosevic M. (2017). Composite Smeared Finite Element—Some Aspects of the Formulation and Accuracy. IPSI Transactions on Advanced Research.

[51] Kojic M., Milosevic M., Kojic N., Koay E.J., Fleming J.B., Ferrari M., Ziemys A. (2018). Mass Release Curves as the Constitutive Curves for Modeling Diffusive Transport within Biological Tissue. Comput. Biol. Med..

[52] Kojic M., Milosevic M., Simic V., Koay E.J., Kojic N., Ziemys A., Ferrari M. (2018). Multiscale Smeared Finite Element Model for Mass Transport in Biological Tissue: From Blood Vessels to Cells and Cellular Organelles. Comput. Biol. Med..

[53] Kojic M., Milosevic M., Simic V., Geroski V., Ziemys A., Filipovic N., Ferrari M. (2019). Smeared Multiscale Finite Element Model for Electrophysiology and Ionic Transport in Biological Tissue. Comput. Biol. Med..

[54] Kojic M. (2018). Smeared Concept as a General Methodology in Finite Element Modeling of Physical Fields and Mechanical Problems in Composite Media. J. Serb. Soc. Comp. Mech..

[55] Milosevic M., Simic V., Milicevic B., Koay E.J., Ferrari M., Ziemys A., Kojic M. (2018). Correction Function for Accuracy Improvement of the Composite Smeared Finite Element for Diffusive Transport in Biological Tissue Systems. Comput. Methods Appl. Mech. Eng..

[56] Milosevic M., Stojanovic D., Simic V., Milicevic B., Radisavljevic A., Uskokovic P., Kojic M. (2018). A Computational Model for Drug Release from PLGA Implant. Materials.

[57] Kojic M., Milosevic M., Ziemys A. (2022). Computational Models in Biomedical Engineering—Finite Element Models Based on Smeared Physical Fields: Theory, Solutions, and Software.

[58] Kojic M., Milosevic M., Simic V., Milicevic B., Terracciano R., Filgueira C.S. (2024). On the Generality of the Finite Element Modeling Physical Fields in Biological Systems by the Multiscale Smeared Concept (Kojic Transport Model). Heliyon.

[59] Forster J.C., Harriss-Phillips W.M., Douglass M.J., Bezak E. (2017). A Review of the Development of Tumor Vasculature and Its Effects on the Tumor Microenvironment. Hypoxia.

[60] Nagy J.A., Chang S.-H., Dvorak A.M., Dvorak H.F. (2009). Why Are Tumour Blood Vessels Abnormal and Why Is It Important to Know?. Br. J. Cancer.

[61] Ganong W.F. (2005). Review of Medical Physiology.

[62] Kurbel S., Flam J. (2007). Interstitial Hydrostatic Pressure: A Manual for Students. Adv. Physiol. Educ..

[63] Boron W.F., Boulpaep E.L. (2017). Medical Physiology.

[64] Open Educational Resources (OER) Services Anatomy and Physiology II. https://courses.lumenlearning.com/suny-ap2/chapter/capillary-exchange.

[65] Boucher Y., Jain R.K. (1992). Microvascular Pressure Is the Principal Driving Force for Interstitial Hypertension in Solid Tumors: Implications for Vascular Collapse. Cancer Res..

[66] Jain R.K., Baxter L.T. (1988). Mechanisms of Heterogeneous Distribution of Monoclonal Antibodies and Other Macromolecules in Tumors: Significance of Elevated Interstitial Pressure. Cancer Res..

