# Supplementary figures and images for: An Insight into Perfusion Anisotropy within Solid Murine Lung Cancer Tumors
Source: Pharmaceutics. 2024 Jul 30;16(8):1009. doi: 10.3390/pharmaceutics16081009 (PMC11360231; doi:10.3390/pharmaceutics16081009)

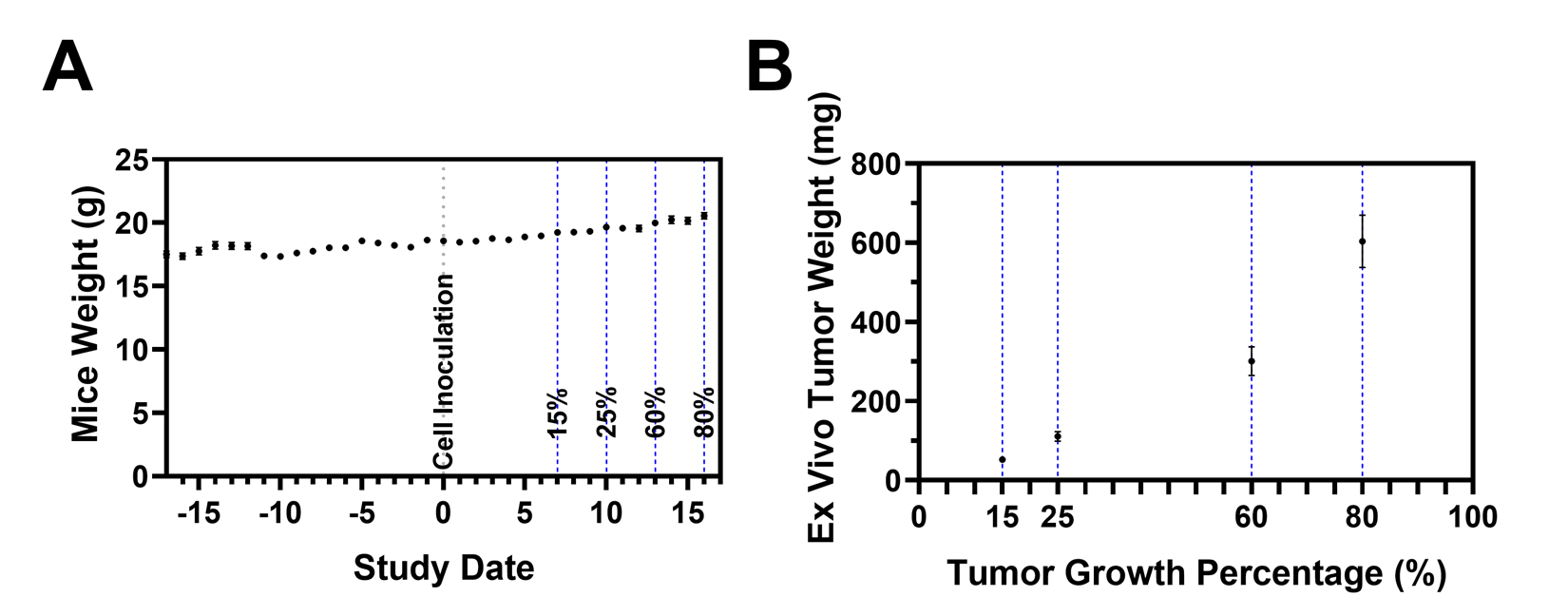

Supplement: Supplementary file 1 [file pharmaceutics-16-01009-s001.zip › Supplemental Figure S1.png]

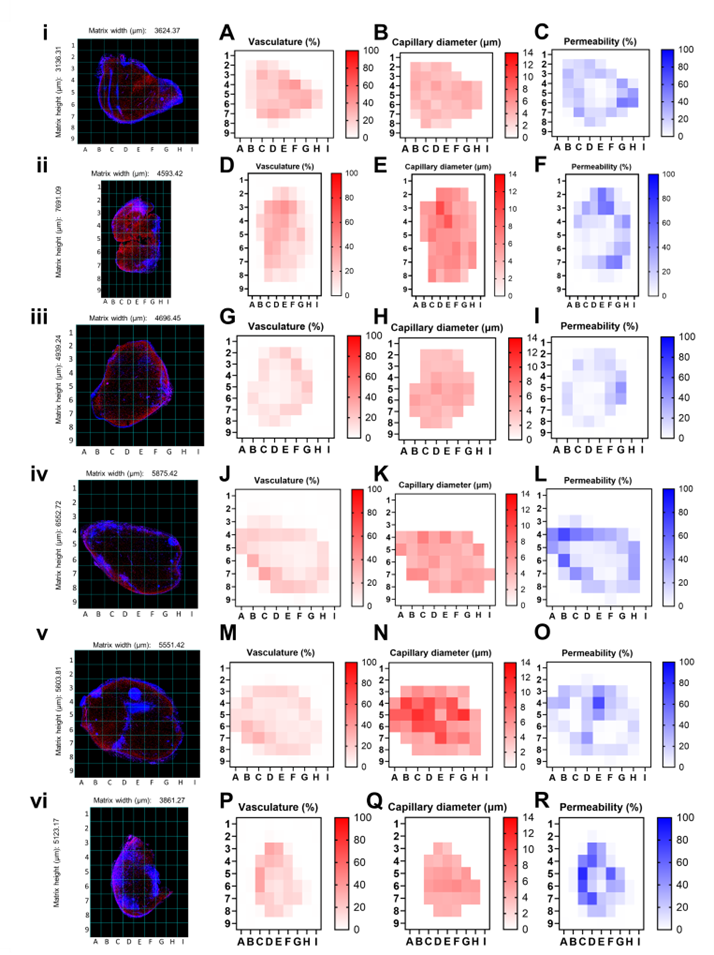

Supplement: Supplementary file 1 [file pharmaceutics-16-01009-s001.zip › Supplemental Figure S2.png]

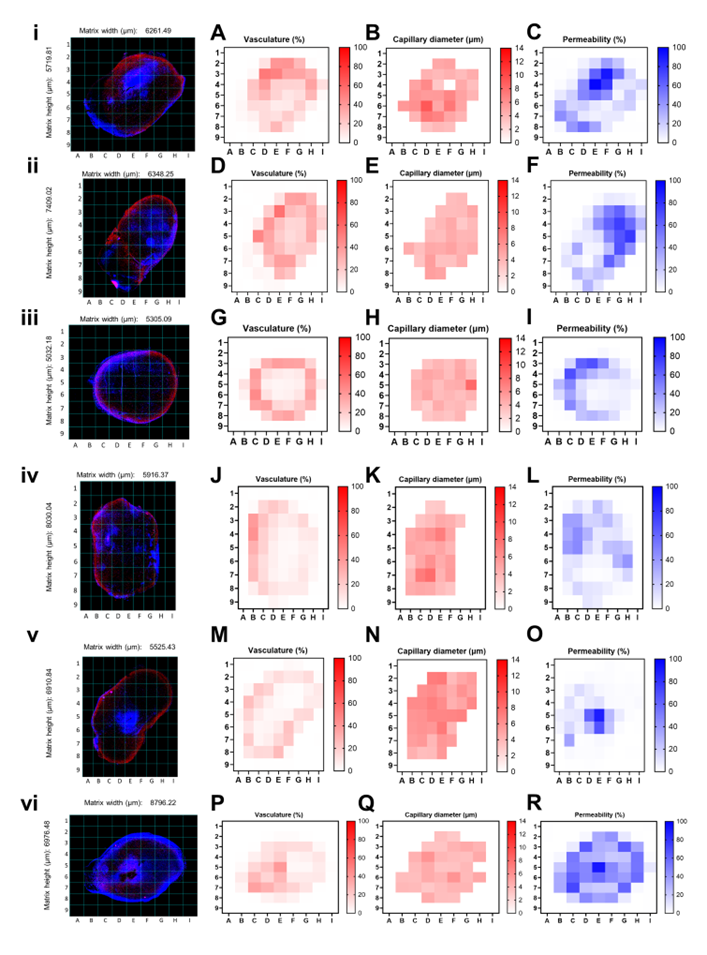

Supplement: Supplementary file 1 [file pharmaceutics-16-01009-s001.zip › Supplemental Figure S3.png]

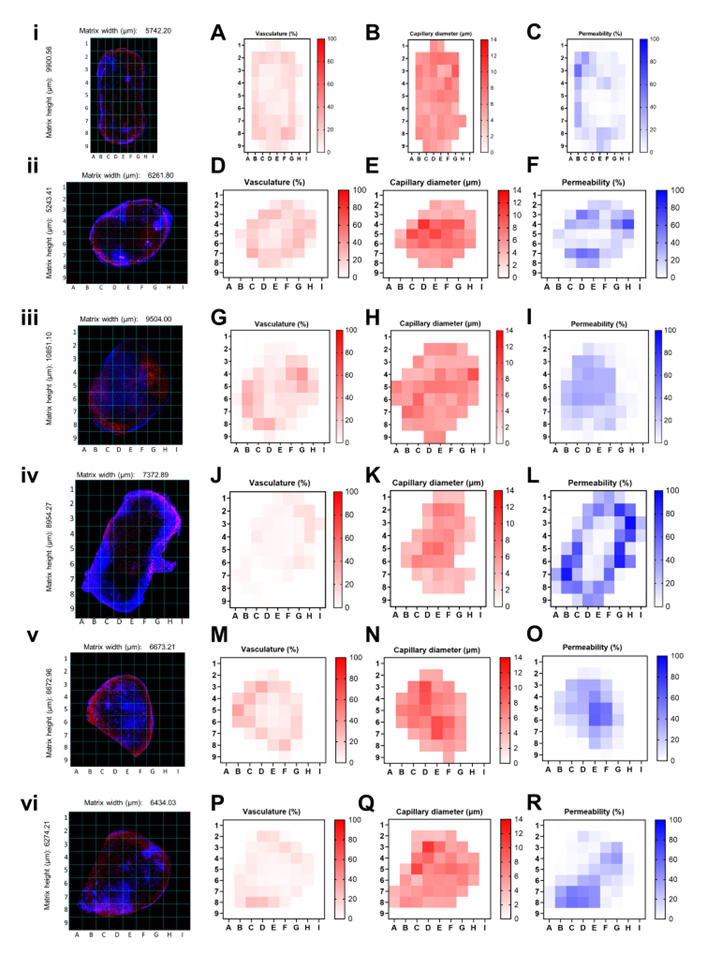

Supplement: Supplementary file 1 [file pharmaceutics-16-01009-s001.zip › Supplemental Figure S4.png]

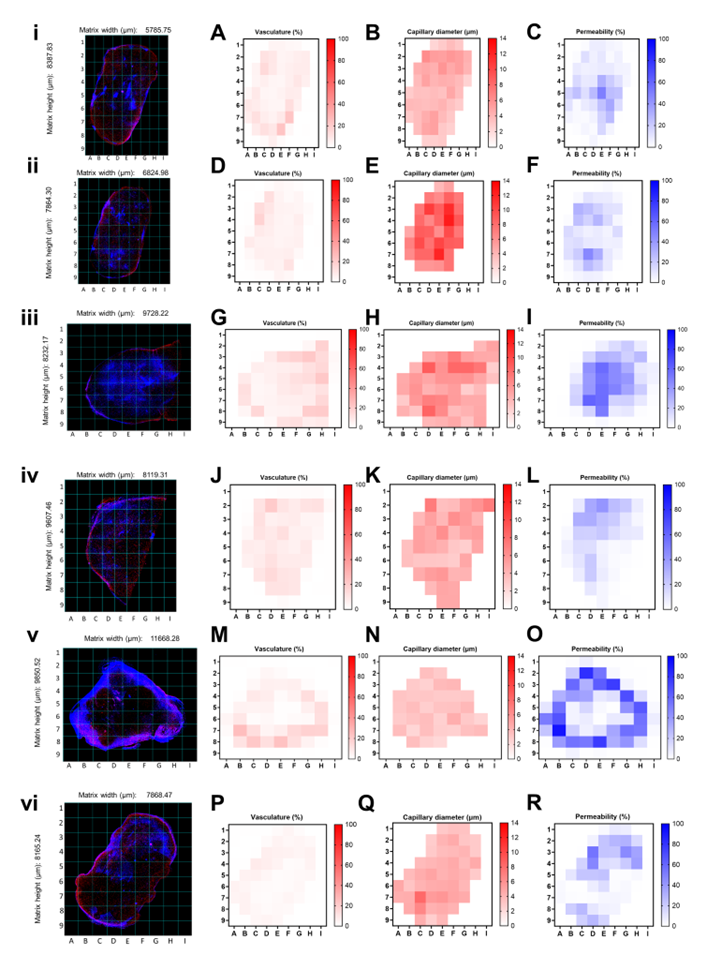

Supplement: Supplementary file 1 [file pharmaceutics-16-01009-s001.zip › Supplemental Figure S5.png]

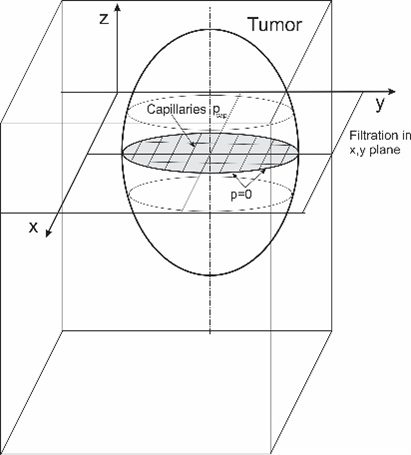

Supplement: Supplementary file 1 [file pharmaceutics-16-01009-s001.zip › Supplemental Figure S6.png]
